# High frequency resistive switching behavior of amorphous TiO_2_ and NiO

**DOI:** 10.1038/s41598-022-16907-8

**Published:** 2022-08-13

**Authors:** Senad Bulja, Rose Kopf, Al Tate, Mark Cappuzzo, Dmitry Kozlov, Holger Claussen, Dirk Wiegner, Wolfgang Templ, Dariush Mirshekar-Syahkal

**Affiliations:** 1 Wireless Communications Laboratory, Tyndall National Institute, 34 Westland Row, Dublin 2, Ireland; 2 Nokia Bell Labs, 600 Mountain Ave., Murray Hill, NJ 07974 USA; 3 Nokia Technology Center, Lise-Meitner-Strasse 7/1, 89081 Ulm, Germany; 4 Nokia Bell Labs Germany, Lorenzstrasse, 10, BW, 70435 Stuttgart, Germany; 5 School of Computer Science and Electronic Engineering (CSEE), University of Essex, Colchester, CO4 3SQ Essex UK

**Keywords:** Electrical and electronic engineering, Electronic devices

## Abstract

Resistive switching (RS) of Transition Metal Oxides (TMOs) has become not only an attractive choice for the development of next generation non-volatile memory, but also as a suitable family of materials capable of supporting high-frequency and high-speed switching needed for the next generation wireless communication technologies, such as 6G. The exact mechanism of RS is not yet clearly understood; however, it is widely accepted to be related to the formation and rupture of sub-stoichiometric conductive filaments (Magnéli phases) of the respective oxides upon activation. Here, we examine the switching behaviour of amorphous TiO_2_ and NiO both under the DC regime and in the high frequency mode. We show that the DC resistance of amorphous TiO_2_ is invariant of the length of the active region. In contrast, the resistance of the NiO samples exhibits a strong dependence on the length, and its DC resistance reduces as the length is increased. We further show that the high frequency switching characteristics of TiO_2_, reflected in insertion losses in the ON state and isolation in the OFF state, are far superior to those of NiO. Fundamental inferences stem from these findings, which not only enrich our understanding of the mechanism of conduction in binary/multinary oxides but are essential for the enablement of widespread use of binary/multinary oxides in emerging non-volatile memory and 6G mm-wave applications. As an example of a possible application supported by TMOs, is a Reflective-Type Variable Attenuator (RTVA), shown here. It is designed to operate at a centre frequency of 15 GHz. The results indicate that it has a dynamic range of no less than 18 dB with a maximum insertion loss of 2.1 dB.

## Introduction

The development of the next generation Non-Volatile Memory (NVM) is widely expected to be driven by non-charge-based mechanisms. This is due to the scaling limitations of charge-based memory, such as Dynamic Random-Access Memory (DRAM). Resistive switching Random Access Memory (RRAM) has drawn significant attention as one of the main contenders for the replacement of DRAM, due to its low fabrication complexity, excellent switching speeds and performance^1–24^. Furthermore, high switching speeds and high dynamic ratios are expected to be the main drivers for the development of the next generation telecommunication systems.

**Figure 1:**
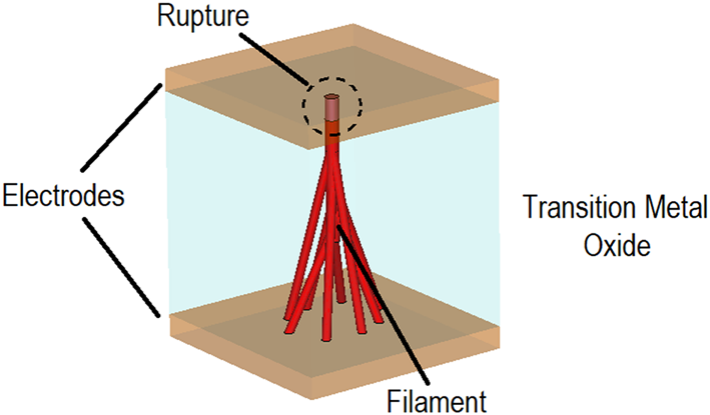
Illustration of filaments in electro-formed crystalline state Transition Metal Oxide. Switching action occurs upon application of DC bias voltage causing establishment of conductive paths.

**Figure 2:**
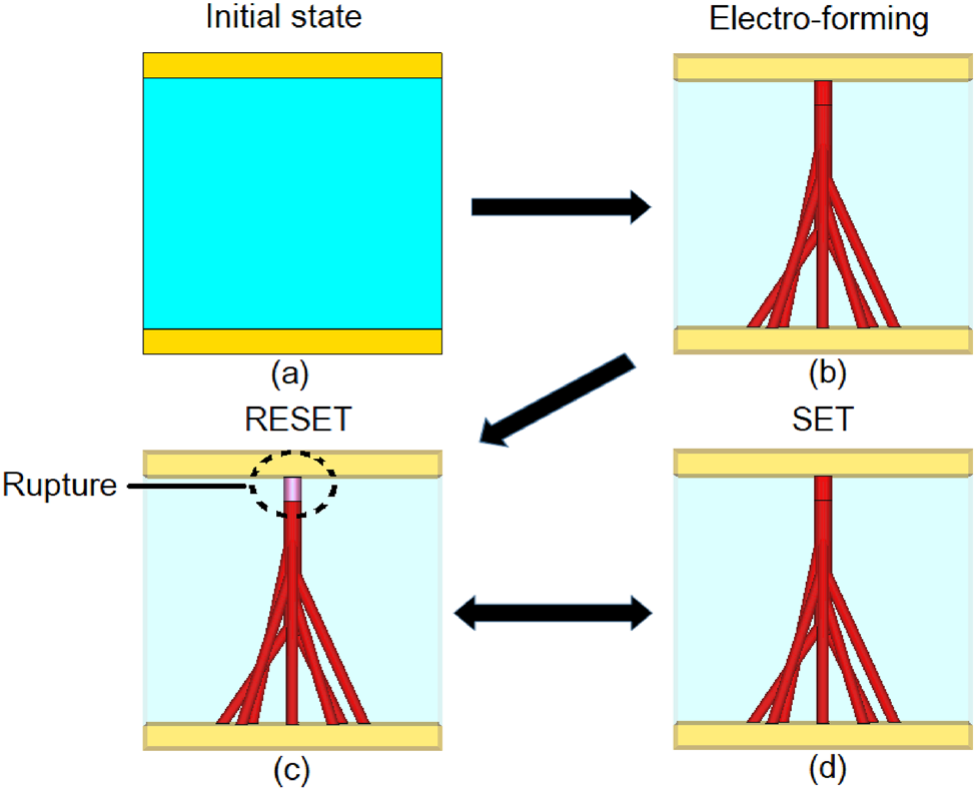
Formation and rupture of conductive filaments (electroforming); (**a**) initial state, (**b**) electro-formation of filament, (**c**) rupture of filament, RESET state and (**d**) reversible SET state.

The physical mechanism behind Resistive Switching (RS) based on Transition Metal Oxides (TMOs) confronts solid-state-physics with the challenge of interpreting the exact nature of the phenomena leading to the reversible transition from the dielectric to conductive states, commonly referred to as Mott transition^13^. The metal–insulator transition in VO_2_ is now accepted to be a (homogeneous) bulk phenomenon^25^, however, the working of RS of the remaining TMOs can be attributed to the formation and rupture of conductive filaments, Figs. 1 and 2, in the interior of the material upon the application of DC bias voltage or temperature elevation. To this end, the filamentary conduction mechanism was experimentally confirmed through in-situ measurements of currents and voltages in Ti_n_O_2n−1_^3,14^ and NiO^26^. The formation of conductive filaments is initiated through the electro-forming process^5,12^ by the application of a DC bias voltage upon the TMO resulting in the creation of sub-stoichiometric conductive filaments (soft dielectric breakdown), Fig. 2b. Upon reversing the DC bias voltage, the stoichiometry of the interior of the TMO is partially restored, resulting in the rupture of the conductive filament, Fig. 2c. Since in this state a TMO cell is not galvanically conductive, it is referred to as the RESET state. The cell can restore its galvanic conductivity by reapplying the DC voltage, shown in Fig. 2d—SET state.

The classical approach to the problem of understanding the switching mechanism of binary/multinary oxides can be erroneous as it is based on treating their physical properties in an analogous way to those of silicon crystals^11^. Indeed, this approach becomes problematic from the outset, due to the fact that the order of defects in binary/multinary oxides is 10–12 orders of magnitude higher than in silicon crystals. On the one hand, this seems to necessitate better quality of crystals, with less defects, however, on the other hand, it needs to be pointed out that RS has been observed in many amorphous TMOs. Therefore, defects in TMOs should not be viewed as undesirable structures for the achievement of “perfect” RS; rather, the goal should lie in forming/engineering “perfect” imperfections, with the aim of achieving high dynamic ratios, high switching speeds and excellent electrical performance. Therefore, any accurate theory or modelling of RS on TMO cannot ignore and indeed needs to consider the role of defects in the material. The defects, depending on their exact nature, can be categorised as “0D” (point defects, such as Schottky and Frenkel disorders), “1D”, “2D” and as “3D” extended defects. Upon activation and the defect agglomeration process these imperfections play a crucial role in the oxide transition into the Magnéli phases, non-stoichiometric conductive filaments. Therefore, the extent of conductivity in the activated state in RS is improved by having the “right” type of defects. From a macroscopic point of view, depending on the stoichiometric nature of the conductive filaments, binary/multinary oxides can be viewed as an n-type semiconductor (such as TiO_2_) or p-type semiconductor (such as NiO) in their respective actuated states.

The use of TMOs in mm-wave applications has been largely limited to the crystalline oxides of vanadium (VO_x_)^15–22^. For example, in Ref.^15^, a 200 nm thick VO_2_ layer, deposited using reactive laser ablation on a Coplanar Waveguide (CPW) to form series and parallel switch configurations, was characterised in the frequency range 5–35 GHz. The switch formed in this way was actuated both thermally (elevating temperature to over 340 K to enable the insulator to metal transition) and electronically (by charge injection). The reported dynamic range is about 25 dB with an insertion loss of about 0.8 dB. The switching speed of VO_2_ is highly dependent on the deposition technique and has been reported to be in the range between 1 ps^17^ to several ns^18,19^. Similar switching speeds have also been recorded for TaO_x_ and ZrO_x_^23,24^. The use of TMOs other than VO_x_ for the purpose of characterisation and use in the context of RF and mm-wave devices has been relatively unexplored, which leaves both a knowledge and application gap. Here, we examine the switching behaviour of amorphous (anatase phase) TiO_2_ and NiO covering both DC and high frequency regimes, to up to 20 GHz. The paper is organised as follows: in “Switch structure”, a switch structure is presented and described. In “DC Results” and “High frequency results” the DC and high frequency characterisation results are presented. “RF switch and attenuator” is dedicated to an application and “Conclusions” to the conclusions.

## Switch structure

**Figure 3:**
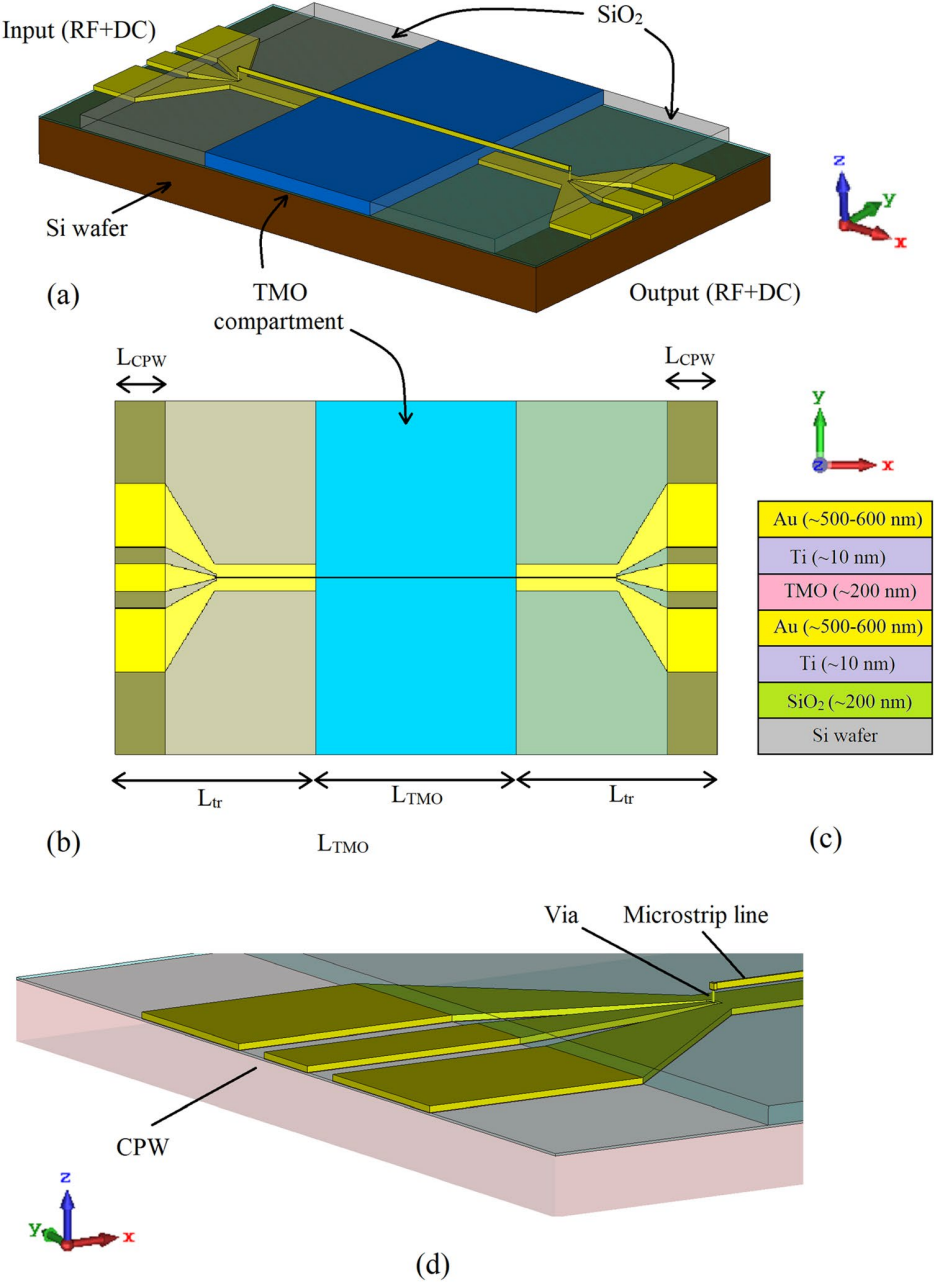
Switch structure: (**a**) perspective view, (**b**) top view, (**c**) material stack, and (**d**) magnified view of CPW-microstrip transition.

**Figure 4:**
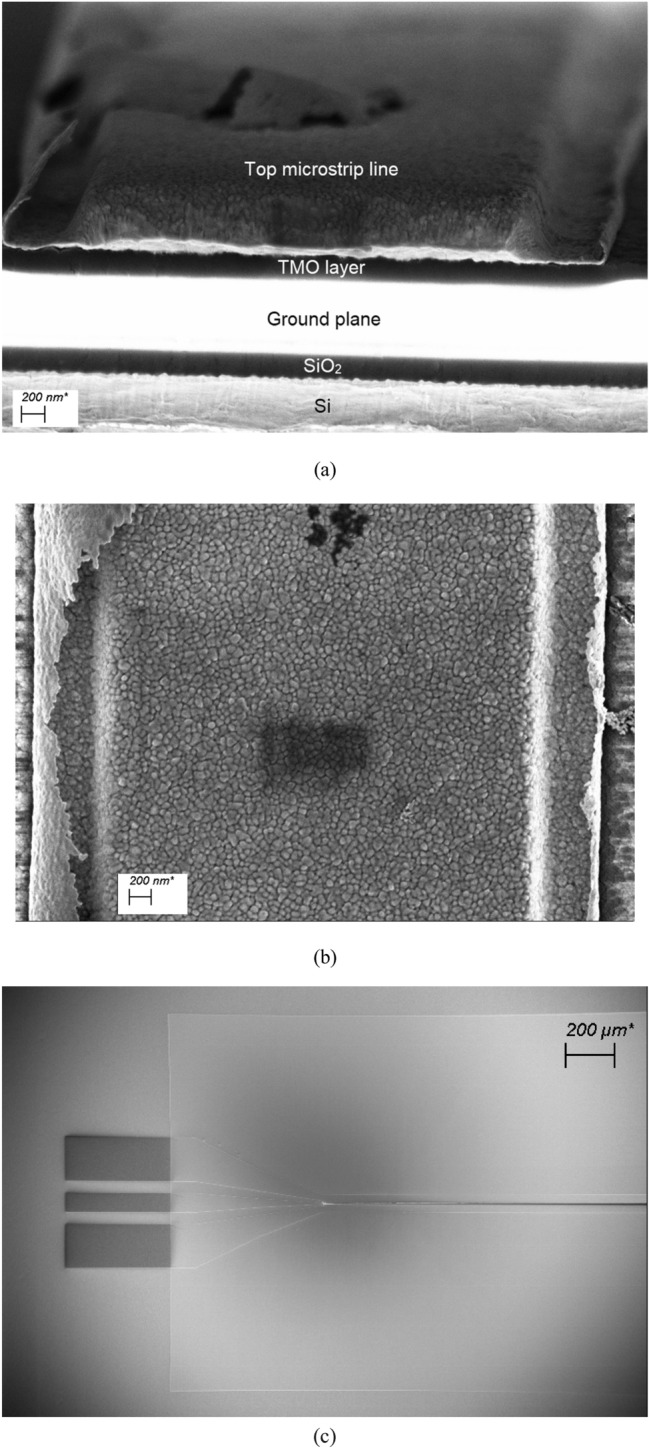
Micrographs of fabricated TMO structures: (**a**) image of cleaved structure showing the top gold line, TMO, and the ground plane obtained using Field Emission Scanning Electron Microscopy (FESEM), (**b**) magnified top view of microstrip line and (**c**) top view of one half of the measurement device. “Curls” on the edges of the microstrip line (**b**) due to lift-off are evident, with an estimated thickness of 40 nm.

The geometry of the proposed switch is illustrated in Fig. 3, and the micrographs of one of the fabricated devices is shown in Fig. 4. The carrier substrate is a 600 µm thick n-doped Si wafer, with a surface resistivity of 100 Ω cm and a dielectric constant of ${\varepsilon }_{r}=11.9$. The Si wafer has 200 nm of thermal oxide grown on top in order to isolate the conductive Si layer from the gold (Au) ground plane deposited on the top of the oxide layer. The thickness of the patterned Au ground plane is around 500–600 nm, with a thin 10 nm Ti layer under the gold layer in order to aid adhesion. The thickness of the Au top electrode is also between 500 and 600 nm, with a 10 nm Ti adhesion layer. The width of the top Au electrode, and hence the anode of the structure of Fig. 3 is W_TMO_ = 8 µm and its length varies from L_TMO_ = 1 mm to L_TMO_ = 5 mm. The top electrode is deposited partially on the SiO_2_ substrate and partially on the TMO layer. The TMO thickness is about 200 nm. The metal and TMO layers were deposited using e-beam evaporation and lift-off. The TMO layers, NiO and TiO_2_, were evaporated from compound sources under an oxygen enriched atmosphere to retain proper stoichiometry. Ellipsometry (Rudolph Auto ELII) and surface profilometry (KLA P7) were used to determine the TMO film refractive index and thickness, respectively. The stoichiometry was then inferred from these measurements.

The SiO_2_ insulating layer between the electrodes in the field was deposited using plasma-enhanced chemical vapor deposition (STS PECVD) and patterned using reactive ion etch (Plasma Therm ICP/RIE). The active TMO region is sandwiched between two Coplanar-Waveguide (CPW) to microstrip transitions of lengths L_tr_ = 2,015 µm, with the length of the exposed pads of L_CPW_ = 400 µm. The CPW-microstrip transition is specially designed for low reflections across the frequency range of interest of 1–20 GHz. It is worth mentioning that instead of the CPW to microstrip transition as pursued here, one could have used an Asymmetric Coplanar Stripline (ACS) to microstrip transition^27^, to achieve the same effect. However, due to the asymmetry of the ACS, the structure formed in this way may render the extraction of the unknown parameters more challenging.

## DC results

**Figure 5:**
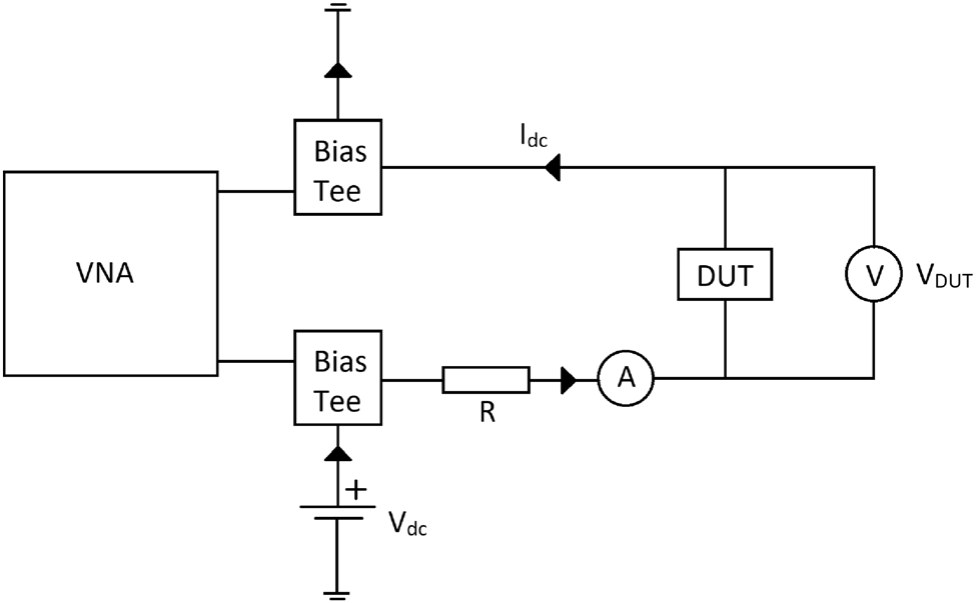
Measurement set-up consisting of a VNA, bias tees, protection resistor, ammeter, voltmeter, and DUT.

**Figure 6:**
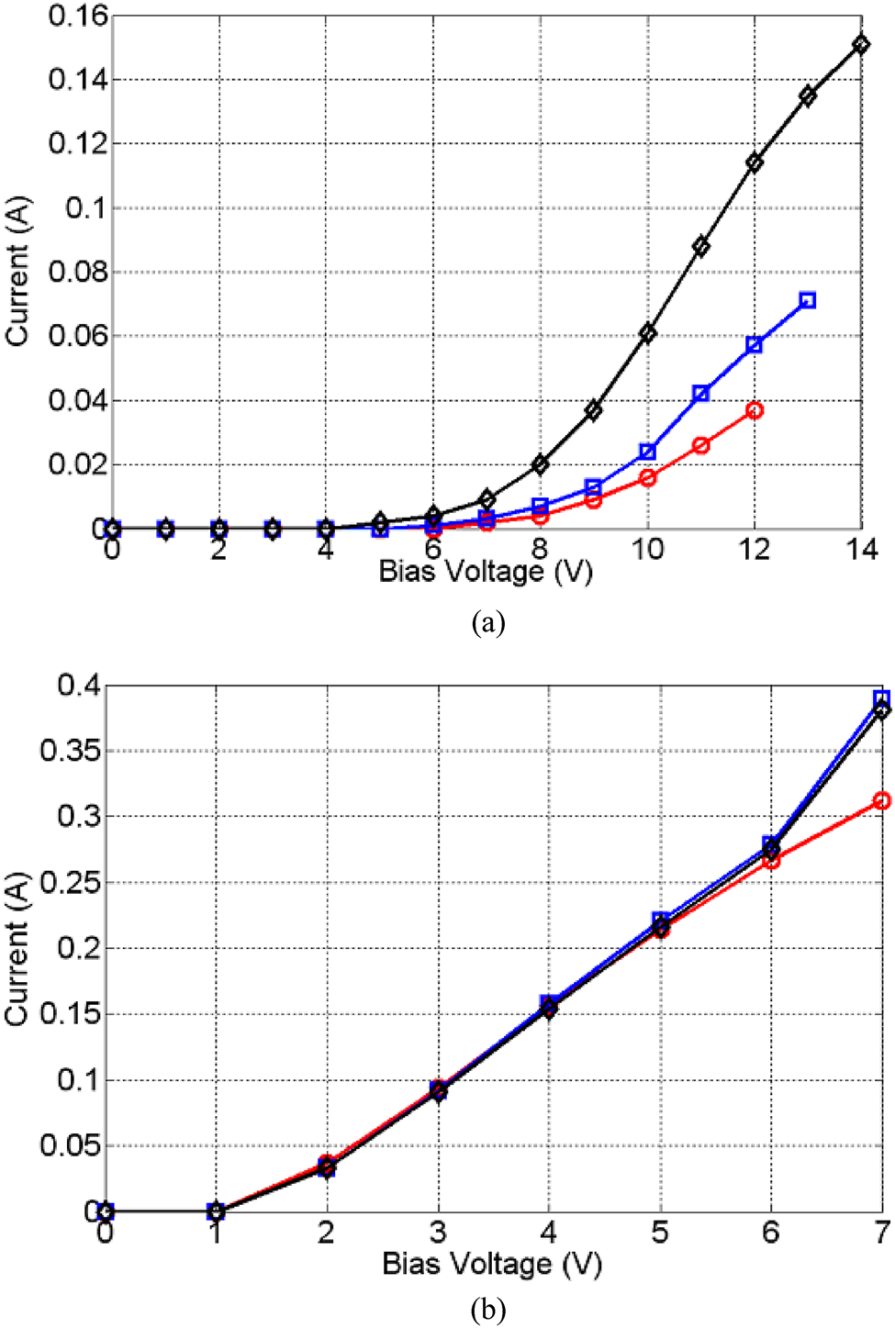
Forward DC bias characteristics of (**a**) NiO and (**b**) TiO_2_ for 1 mm (red circles), 2 mm (blue squares) and 5 mm (black diamond) length.

Figure 5 shows the measurement set-up used for the measurement of DC and high frequency performances. Here, a Vector Network Analyzer (VNA) is connected to the Device Under Test (DUT), (shown in Fig. 3), through bias tees, enabling DC biasing of the structure. During the DC test the VNA is kept switched off and voltages and currents are measured by external Volt and Amp meters, Fig. 5. Since at the onset of the Insulator-to-Metal Transition (formation of Magnéli phases), the current through the device can exhibit a run-away behavior, a current limiting resistor of R = 600 Ω is added in the set-up. Several devices fabricated using TiO_2_ and NiO with active region lengths of 1 mm, 2 mm and 5 mm were tested. To this end, the DC voltage was gradually increased and the DC current was monitored, Fig. 6. With reference to this figure, it is important to note that the DC bias voltages applied to the NiO and TiO_2_ samples are not identical. Here, it was empirically observed that the maximum DC bias voltage that can be applied to the TiO_2_ samples is independent on their length and stands at 7 V. Increasing the DC bias voltage beyond this value results in the samples’ destruction, probably due to Joule heating. The situation is somewhat different for the NiO samples. In this case, the maximum DC bias voltage that can be applied to the samples is empirically determined to depend on the sample’s length. It stands at 12 V, 13 V and 14 V for the NiO lengths of 1 mm, 2 mm and 5 mm, respectively. It was further observed that even though the DC resistance in the OFF state remained high regardless of the TMO type and its length, the full-ON state resistance is dependent on the type of the TMO. For instance, when the lengths of the active NiO regions are 1 mm, 2 mm and 5 mm, the full-ON measured DC resistances are 300 Ω, 185 Ω and 93 Ω, respectively. In contrast, for the same lengths of the active regions, the full-ON DC resistance of the TiO_2_ samples varies from 18.4 to 21.8 Ω, which is relatively small. This behavior is insightful, since it points to two different conduction mechanisms, one working in NiO another one in TiO_2_. It can be speculated that in the amorphous NiO samples, the size of the sub-stoichiometric conductive filaments is smaller than their counterparts in TiO_2_, and, also, that the number of filaments scales with length (of the NiO region). Regarding the TiO_2_ samples, it appears that within their active domains, only a few dominant conductive filaments are established, rendering the DC resistance of TiO_2_ samples relatively insensitive to their length.

**Figure 7:**
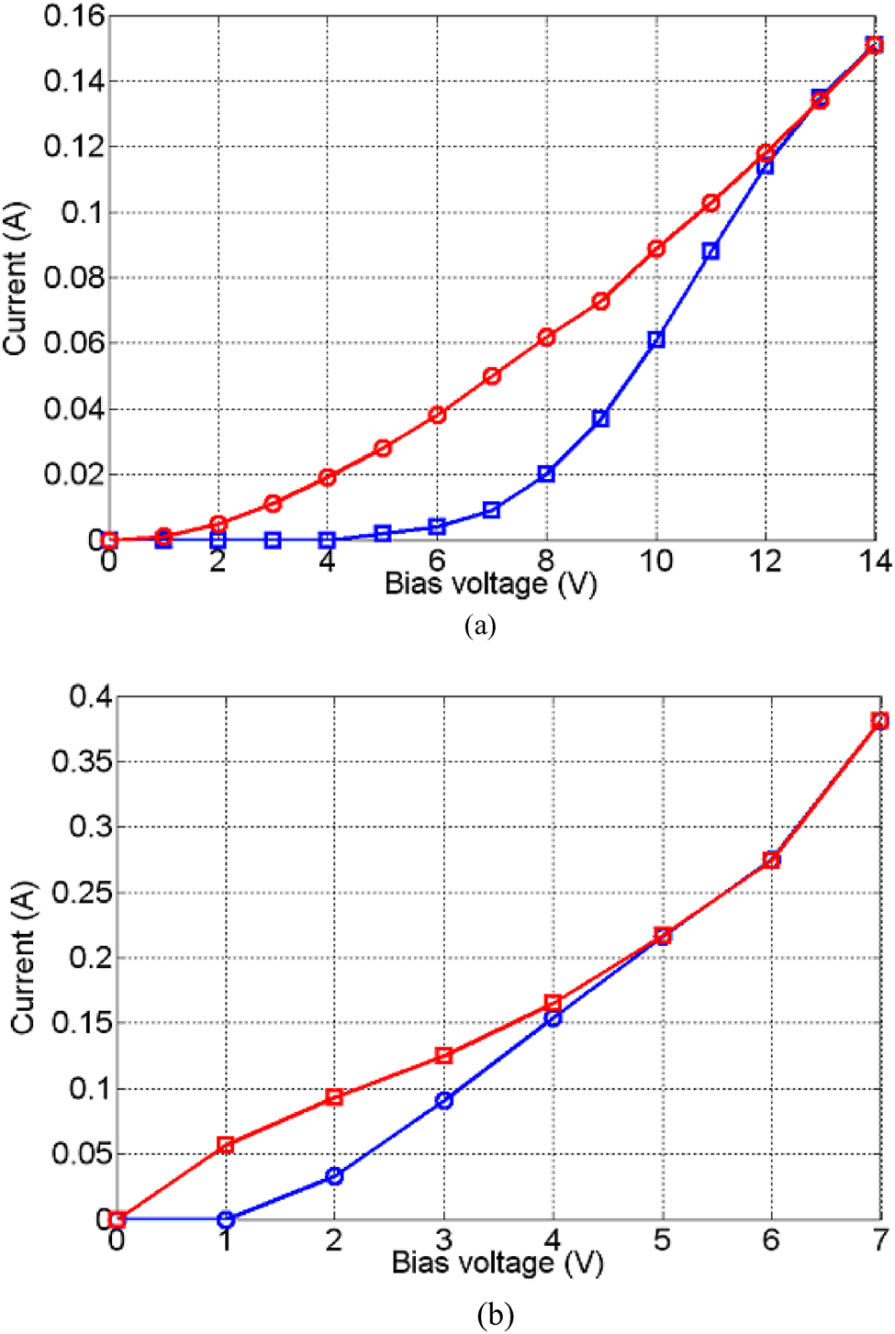
DC cycling of 5 mm long active regions; (**a**) NiO and (**b**) TiO_2_: (blue circles) cycling up and (red squares) cycling down (showing hysteresis).

Next, the effect of DC cycling (repeatedly driving the test cells into the ON and OFF states) on the behavior of the proposed amorphous oxides was investigated and the results are shown, Fig. 7. The lengths of the active samples for both cases are 5 mm. Here, both devices were cycled several times and no difference in the response was recorded. As can be seen, no DC current runaway was detected for both samples, however, the recorded hysteresis is in line with previous literature findings^1–24^.

## High frequency results

**Figure 8:**
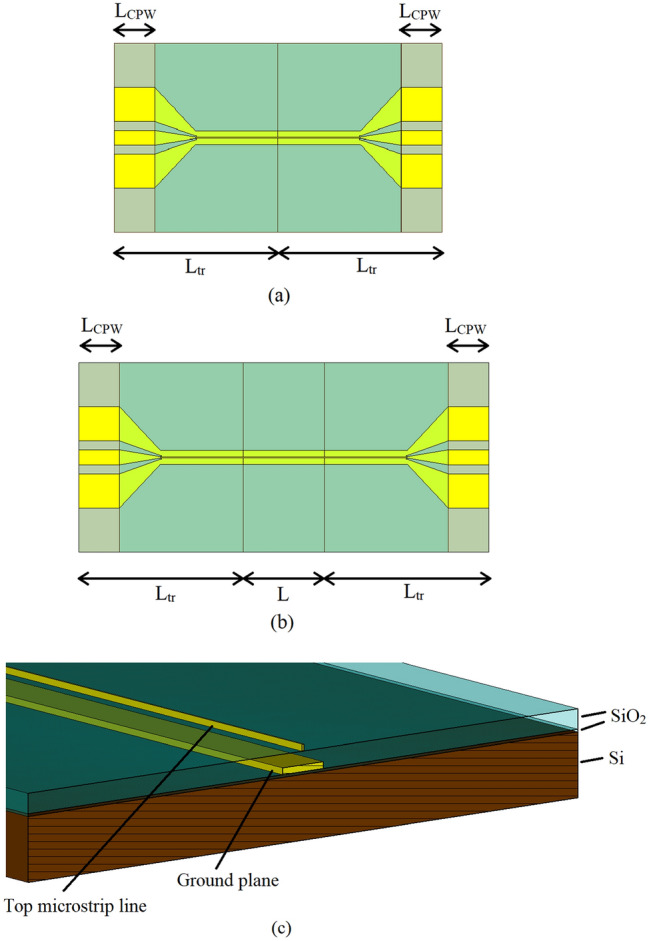
The two passive structures used in de-embedding; (**a**) Through standard, (**b**) Line standard, and (**c**) cross-section of passive devices.

To assess the performance of the TMO switches at high frequencies, initially the influences of the two CPW-microstrip transitions evident in Fig. 3 need to be extracted from the measured scattering parameters of the switch structure. The scattering parameters of the transitions can be obtained by deploying a two-tier, thru-line technique^28^, which relies on the measurements of two passive structures shown in Fig. 8.

**Figure 9:**
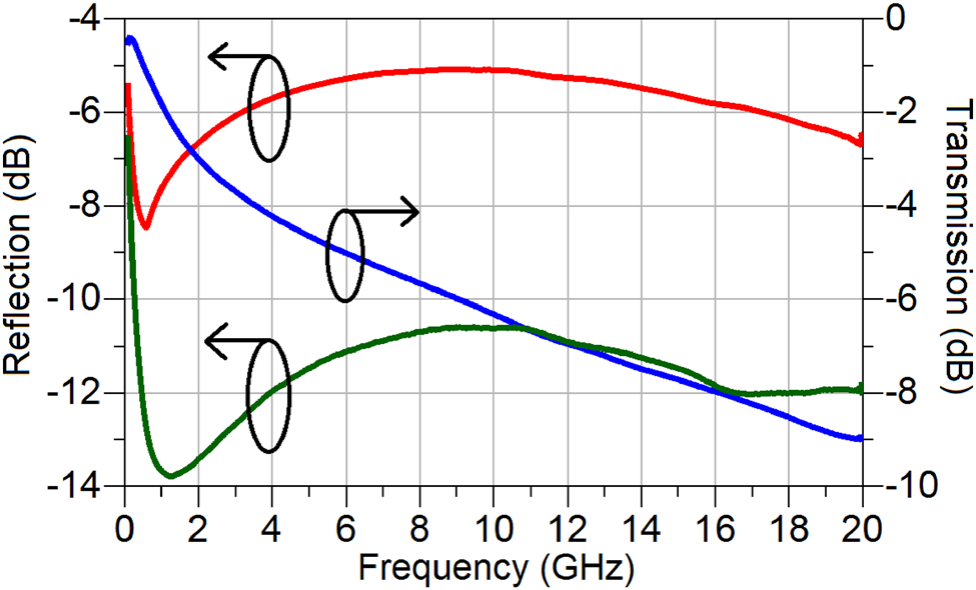
Extracted scattering parameters of the CPW-microstrip transition; S11 (red, as seen from CPW side), S22 (green, as seen from microstrip line side) and S21 (blue).

The first passive structure contains two CPW-to-microstrip transitions (as appears in Fig. 3) connected back-to-back, termed Thru (T). The second passive structure also contains the two-transitions connected back-to-back, but through a length of a line, termed Line (L). None of these two calibration standards, the Thru and the Line, are exposed to the active (TMO) material, and indeed, they are fabricated on the passive (SiO_2_) material, which is common to the microstrip line sections of both CPW-to-microstrip transitions. The length of the Line (L) standard is chosen to be 1 mm, and, as will be seen later, it is selected to be the same as the length of the active (TMO) cell. The scattering parameters of the two standards were measured using a probe station and a Vector Network Analyser (VNA) with no DC bias voltage supplied through the bias tee. The scattering parameters extracted for each transition are shown in Fig. 9. As evident, the reflection coefficient at the input port (CPW side) is better than − 5 dB, while the output reflection coefficient (microstrip line side) is better than − 10 dB. The transmission coefficient of the transition, as shown in Fig. 9 experiences a high level of attenuation. This is understandable, since both the ground plane and microstrip line are electrically very thin, being approximately 0.37 skin depths (δ) thick even at the highest frequency of operation of 20 GHz. At 1 GHz, the electrical thickness is even smaller and is equal to 0.08δ. This renders the characteristic impedance of the CPW and microstrip line highly frequency dependent, which adversely affects impedance matching and transmission loss.

Having determined the scattering parameters of the transitions, it is now possible to consider their effects and to obtain the switching characteristics of the amorphous NiO and TiO_2_ samples accurately. The transmission-line matrix of the active region in Fig. 3, containing NiO or TiO_2,_ can be found from

1 $${[T]}_{TMO}={[T]}_{trans}^{-1}\cdot {\left[T\right]}_{meas}\cdot {\left[T\right]}_{re{v}\_{trans}}^{-1}.$$

where, ${[T]}_{meas}$, represents the transmission matrix of the entire structure shown in Fig. 3, after converting the measured scattering parameters to the transmission parameters. Further, ${[T]}_{trans}^{-1}$ and ${[T]}_{rev\_trans}^{-1}$ represent the inverse transmission matrices of the two input and output CPW-microstrip transitions arranged in the forward and reverse positions, respectively.${[T]}_{TMO}$ denotes the extracted transmission matrix of the active TMO region, which includes the effect of any impedance mismatch at the two junctions between the microstrip line deposited on the SiO_2_ substrate and the active, TMO region. This is manifested by the existence of reflections emanating from this boundary. The effect of the unwanted reflections makes it difficult to adequately compare the extracted switch performances of the two TMOs and, therefore, it needs to be taken into account. The process of transforming the extracted ${[T]}_{TMO}$ matrix containing the unwated reflections into a reflection-less transmission matrix is described below.

**Figure 10:**
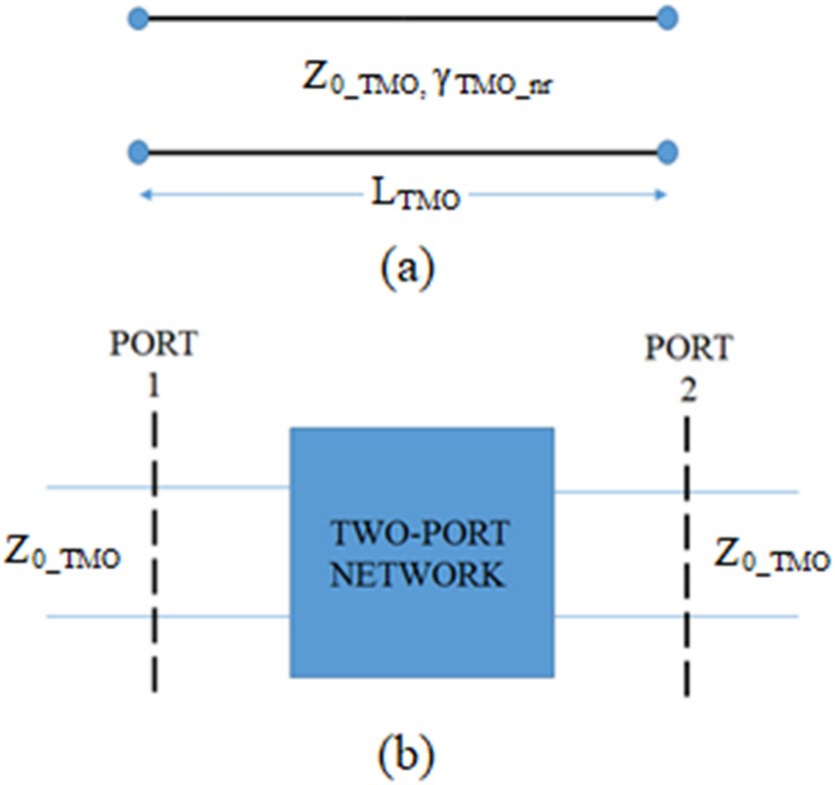
Standard transmission line; (**a**) Transmission line of length L_TMO_ and (**b**) a two-port network terminated in Z_0_TMO_.

The ABCD parameters of a perfectly impedance matched transmission line of Fig. 10a, containing the unknown propagation constant γ_TMO_nr_ and characteristic impedance Z_0_TMO,_ γ_TMO_nr_ are given below

2 $$\left(\begin{array}{cc}{A}_{TM{O}_{nr}}& { B}_{TM{O}_{nr}}\\ {C}_{TM{O}_{nr}}& { D}_{TM{O}_{nr}}\end{array}\right) =\left(\begin{array}{cc}\mathrm{cosh}\left({\gamma }_{TMO\_nr}{\cdot L}_{TMO}\right)& {Z}_{0\_TMO}\mathrm{sinh}\left({\gamma }_{TMO\_nr}{\cdot L}_{TMO}\right)\\ \frac{\mathrm{sinh}({\gamma }_{TMO\_nr}\cdot {L}_{TMO})}{{Z}_{0\_TMO}}& \mathrm{cosh}({\gamma }_{TMO\_nr}{\cdot L}_{TMO})\end{array}\right).$$

where, L_TMO_ is the length of the active, TMO region. A simple transformation of (2) into scattering parameters yields.

3 $$\left(\begin{array}{cc}{S}_{11\_TMO\_nr}& { S}_{12\_TMO\_nr}\\ {S}_{21\_TMO\_nr}& {S}_{22\_TMO\_nr}\end{array}\right)= \left(\begin{array}{cc}0& {e}^{-{\gamma }_{TMO\_nr}\cdot {L}_{TMO}}\\ {e}^{-{\gamma }_{TMO\_nr}{\cdot L}_{TMO}}& 0\end{array}\right).$$

On the other hand, the ABCD parameters of the active region expressed through the general form of the ${[T]}_{TMO}$ matrix, shown in Fig. 10b, are

$${A}_{TMO}=\frac{1}{2}\left({T}_{{11}_{TMO}}+{T}_{{22}_{TMO}}+{T}_{{21}_{TMO}}+{T}_{{12}_{TMO}}\right).$$

$${B}_{TMO}=\frac{{Z}_{0\_TMO}}{2}\left({T}_{11\_TMO}-{T}_{22\_TMO}+{T}_{21\_TMO}-{T}_{12\_TMO}\right).$$

$${C}_{TMO}=\frac{1}{2{Z}_{\_TMO}}\left({T}_{11\_TMO}-{T}_{22\_TMO}-{T}_{21\_TMO}+{T}_{12\_TMO}\right).$$

4 $${D}_{TMO}=\frac{1}{2}\left({T}_{11\_TMO}+{T}_{22\_TMO}-{T}_{21\_TMO}-{T}_{12\_TMO}\right).$$

In order that the transmission line formed in this way is reflection-less, the propagation constant γ_TMO_nr_ of (2) also needs to satisfy (4). By equating ${A}_{TM{O}_{nr}}$ and ${A}_{TMO}$, the propagation constant of the reflection-less line can be found from.

5 $$\mathrm{cosh}\left({\gamma }_{TMO\_nr}{L}_{TMO}\right)= \frac{\left({T}_{11\_TMO}+{T}_{22\_TMO}\right)+\left({T}_{12\_TMO}+{T}_{21\_TMO}\right)}{2}.$$

Equation (5) can be further simplified by considering that the active region described by the ${[T]}_{TMO}$ is symmetrical, i.e.

6 $${T}_{11\_TMO}\cdot {T}_{{22}_{TMO}}-{T}_{{21}_{TMO}}\cdot {T}_{{12}_{TMO}}=1 \; \mathrm{and}\; {T}_{12\_TMO}=-{T}_{{21}_{TMO}}.$$

which yields the following expression for the propagation constant of the reflection-less transmission given by (2) and (3)

7 $${\gamma }_{TMO\_nr}{\cdot L}_{TMO}={cosh}^{-1}\left(\frac{{T}_{11\_TMO}^{2}-{T}_{21\_TMO}^{2}+1}{{2T}_{11\_TMO}}\right).$$

or expressed via the scattering parameters of the ${[T]}_{TMO}$ matrix,

8 $${\gamma }_{TMO\_nr}\cdot {L}_{TMO}={cosh}^{-1}\left(\frac{1+{S}_{{11}_{TMO}}^{2}-{S}_{{21}_{TMO}}^{2}}{{2S}_{{21}_{TMO}}}\right).$$

Such a reflectionless line is then fully described by (3) and the performances of the active regions of NiO and TiO_2_ can be adequately compared by using the transmission coefficient only, given by:

9 $${S}_{21\_TMO\_nr}={e}^{-{\gamma }_{TMO\_nr}\cdot {L}_{TMO}}.$$

**Figure 11:**
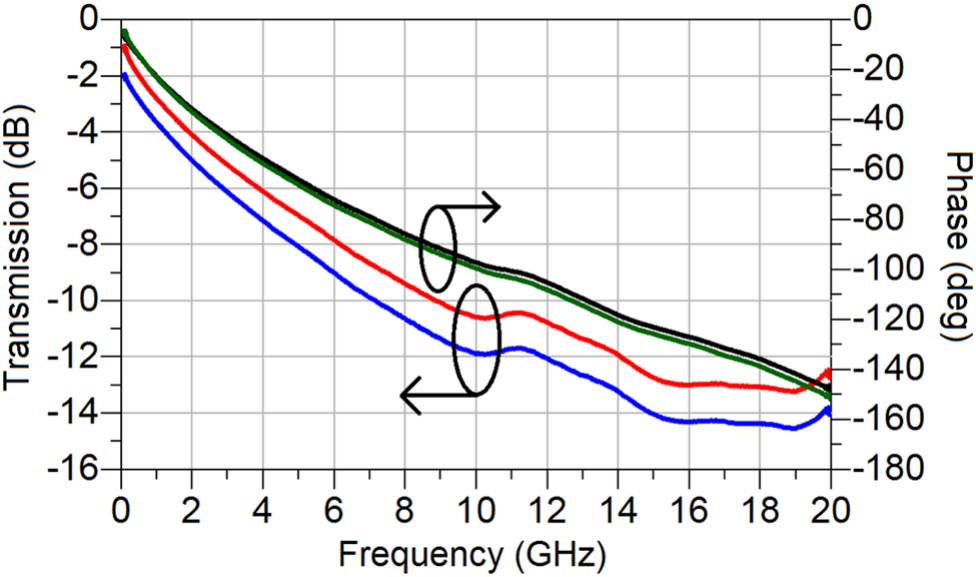
Extracted transmission coefficients of amorphous NiO; red (OFF state, magnitude), blue (ON state 12 V, magnitude), black (OFF state, phase) and green (ON state 12 V, phase).

**Figure 12:**
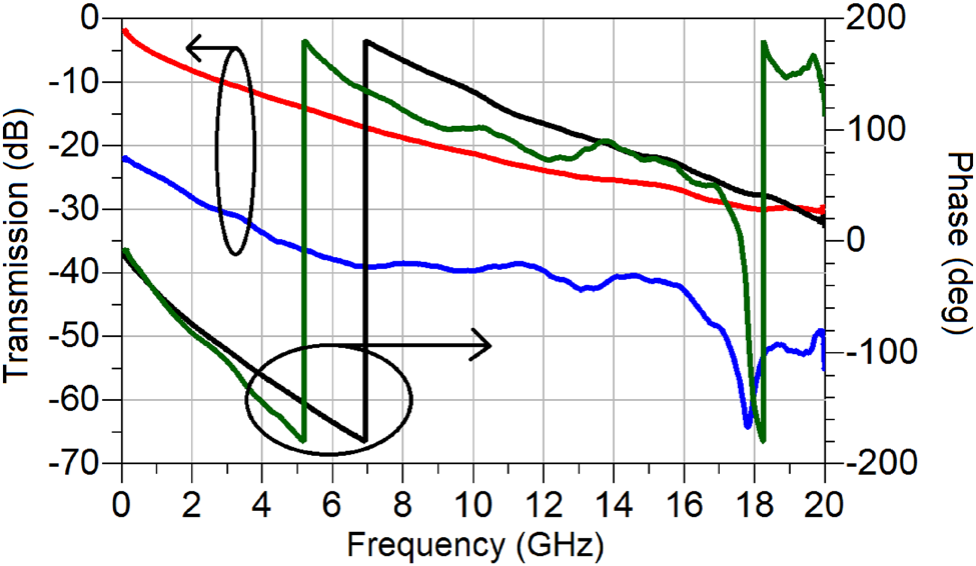
Extracted reflection-less transmission coefficients of amorphous TiO_2_; red (OFF state, magnitude), blue (ON state 7 V, magnitude), black (OFF state, phase) and green (ON state 7 V, phase).

The extracted reflectionless transmission coefficients of the amorphous NiO and TiO_2_ for the same L_TMO_ = 1 mm length of the active regions are plotted in Figs. 11 and 12, respectively. It is evident from these two figures that the transmission losses for both TMOs are rather high, however, that is understandable in the context of electrically thin conductors, as explained earlier. For the case of the NiO based TMO, the maximum difference between the OFF and ON states is about 2 dB, while for the case of TiO_2_ based TMO, the difference between the OFF and ON states is no less than 15 dB over the frequency range 10 MHz to 20 GHz. It is further interesting to note that the switching of the amourphous NiO is accompanied by a small change in the transmission phase (maximum of 5° at 20 GHz), whereas switching between states in TiO_2_ shows that the transmission coefficient bears a much larger phase change (maximum of 90° at 20 GHz). Therefore, this phenomenon deserves further investigation.

Next turning to the extraction of the propagation constant,${\gamma }_{TMO\_nr}$, in (9), which contains not only the information about the TMO, but it is distorted by the parasitic EM propagation through the electrically thin microstrip line and the dielectric directly above it (air in this case). If viewed in a stricter sense, the dielectric property of the TMO structure, Fig. 3, needs to be represented by a second rank, in-plane-spatially dependent tensor, i.e.

10 $$\left(\begin{array}{c}{D}_{x}\\ {D}_{y}\\ {D}_{z}\end{array}\right)=\left(\begin{array}{ccc}{\overline{\varepsilon }}_{\parallel }& 0& 0\\ 0& {\overline{\varepsilon }}_{\parallel }& 0\\ 0& 0& {\overline{\varepsilon }}_{\perp }\end{array}\right)\left(\begin{array}{c}{E}_{x}\\ {E}_{y}\\ {E}_{z}\end{array}\right).$$

Here, ${\overline{\varepsilon }}_{\parallel }$ is the x or y-direction complex dielectric characteristics (constant) of the TMO layer, while ${\overline{\varepsilon }}_{\perp }$ represents the z-direction complex dielectric characteristics of the TMO. Since the dominant propagation mode in the proposed structure is Quasi Transversal Electro-Magnetic (QTEM) wave, there exists little field variation in the direction of x- and y-axis, Fig. 3. Further, as the ratio of the width of the top electrode of the TMO structure of Fig. 3 to the TMO structure height is very large (about 40), it can be reasonably assumed that the EM fields are primarily confined to the area under the top electrode with little variation in the tangential plane. Therefore, EM propagation can be fully described by the knowledge of ${\overline{\varepsilon }}_{\perp }$, which can be assumed to be homogeneously distributed in the volume under the top electrode. As such, it would be pertinent now to refer to ${\overline{\varepsilon }}_{\perp }$ as ${\overline{\varepsilon }}_{r}$ of the TMO. In other words, the TMO structure can be described by a single, composite dielectric characteristic, where ${\overline{\varepsilon }}_{\perp }={\overline{\varepsilon }}_{r}={\varepsilon }_{r}^{^{\prime}}+j{\varepsilon }_{r}^{"}$ and $\mathrm{tan}\left(\delta \right)= {\varepsilon }_{r}^{\prime\prime}/ {\varepsilon }_{r}^{\prime}$. However, it needs to be borne in mind that due to the existence of conductive Magnéli phases in the medium surrounded by insulating stoichiometric TMO in the ON state, the extracted high frequency values of ${\overline{\varepsilon }}_{r}$ are expected to exhibit a mixed high dielectric-metal behavior.

Even with these assumptions, the analytical extraction of the unknown dielectric parameters of TMO from the complex propagation characteristics for a standard microstrip is impossible, due to the difficulties of separating the loss contributions of the conductive and dielectric parts. In the present case, this is further complicated by the fact that the bottom and top electrodes are electrically thin in the frequency region from 1 to 20 GHz. The thin conductors manifest themselves not only in the form of increased losses in the structure, but they also affect the real part of the dielectric permittivity. Furthermore, as shown in Fig. 4, the lift-off process for the top gold layer (microstrip line) is not optimized, which has led to the creation of edge curling. The average thickness of the curls is about 40 nm, resulting in the creation of a waveguide effect, macroscopically manifesting itself as an additional loss mechanism. Therefore, the extraction of the dielectric parameters is performed numerically, using a Finite Integration Technique, implemented in a commercially available full-wave simulator, CST^29^, where an average edge curling dimension of 40 nm is assumed to extend along the length of the TMO structure. Here, the extracted values of the complex propagation constant, ${\gamma }_{TMO\_nr}$, are computationally matched to the propagation constant predicted by simulations across the entire frequency range using, among other parameters, the dielectric characteristics of the TMOs as optimisation parameters. For the purpose of extraction, it is assumed that the unknown characteristics of TMOs are dielectric in nature in both OFF and ON states and, therefore, the extraction is carried out with respect to ${\varepsilon }_{r}^{^{\prime}}$ and ${\varepsilon }_{r}^{"}$ of the TMO material. At a subsequent step, and in order to reflect the phase changing nature of TMO materials, the losses in the OFF state are represented in terms of the loss tangent, whereas the ON state losses are described using the equivalent electrical conductivity, to take into account the transformation of the materials into a conductive state. The values of the loss tangents are obtained using the ratio of ${\varepsilon }_{r}^{"}$ to ${\varepsilon }_{r}^{^{\prime}}$.

**Figure 13:**
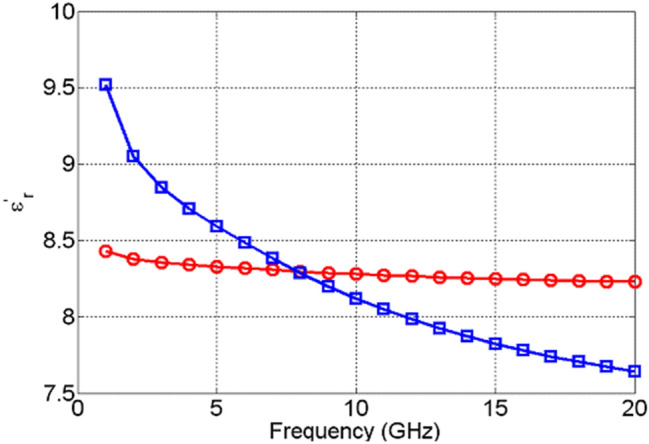
Extracted ${\varepsilon }_{r}^{^{\prime}}$ of NiO in OFF state (red circles) and in ON state (blue squares).

**Figure 14:**
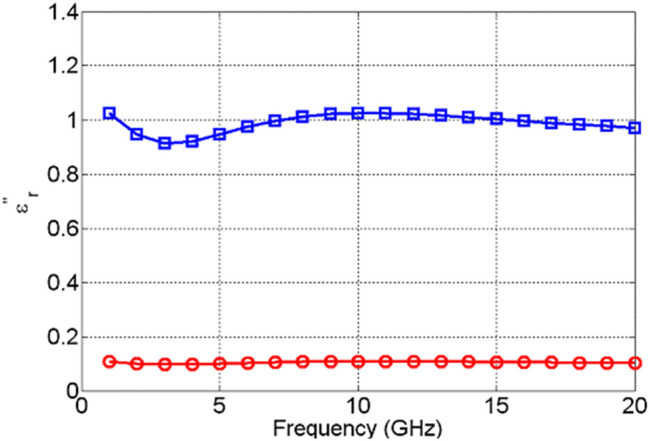
Extracted ${\varepsilon }_{r}^{"}$ of NiO in OFF state (red circles) and ON state (blue squares).

**Figure 15:**
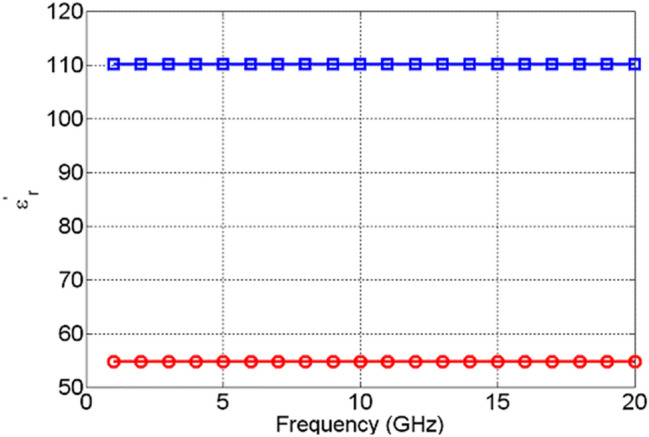
Extracted ${\varepsilon }_{r}^{^{\prime}}$ of TiO_2_ OFF state (red circles) and ON state (blue squares).

**Figure 16:**
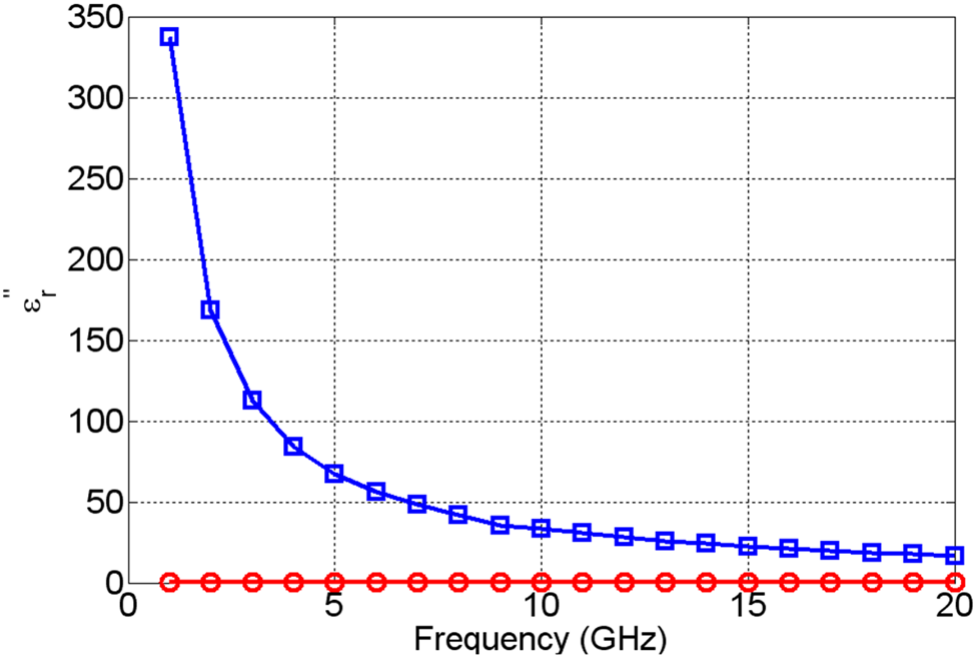
Extracted ${\varepsilon }_{r}^{"}$ of TiO_2_ in OFF state (red circles) and in ON state (blue squares).

**Figure 17:**
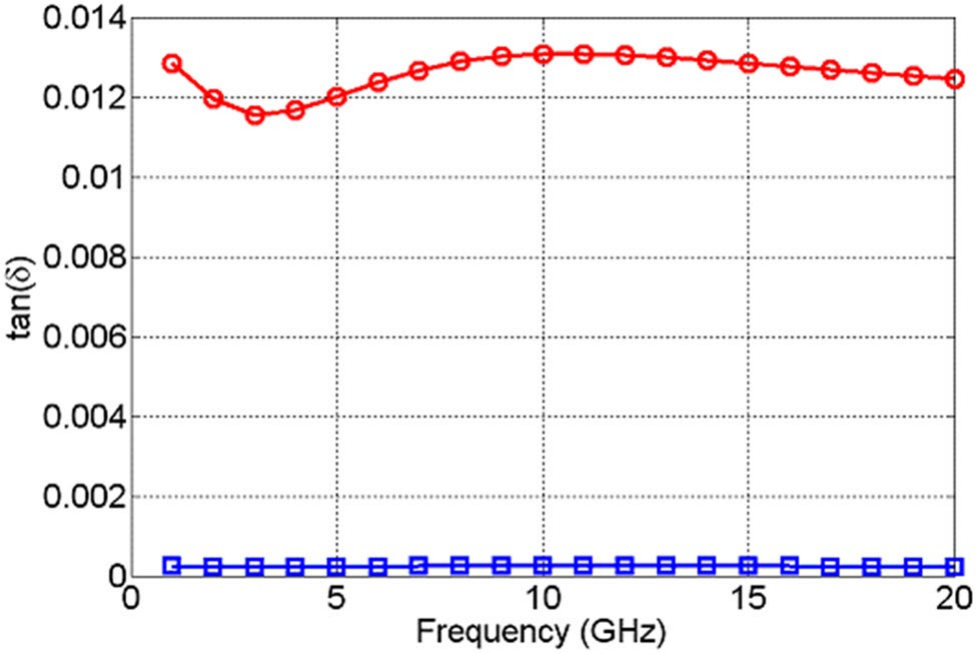
Extracted loss tangents of NiO (red circles) and TiO_2_ in OFF state (blue squares).

**Figure 18:**
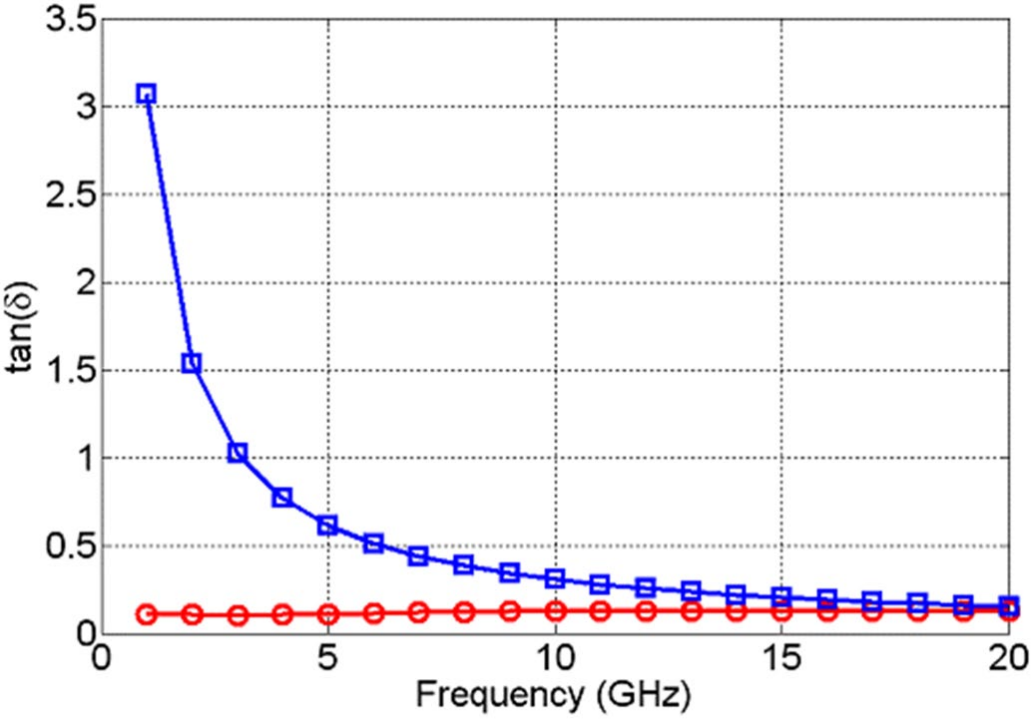
Extracted loss tangents of NiO (red circles) and TiO_2_ in ON state (blue squares).

The change in the values of the real part of the dielectric permittivity, ${\varepsilon }_{r}^{^{\prime}}$, of NiO, Fig. 13, upon actuation is found not only to be small, but it also exhibits excursions both above and below the values it attained prior to actuation. This is due to the high losses that NiO acquires upon actuation, shown in Fig. 14, which are not followed by a significant change in the dielectric permittivity. TiO_2_, on the other hand, exhibits a significant change in the real part of dielectric permittivity upon actuation—over 200%, as shown in Fig. 15. Furthermore, TiO_2_ also exhibits a much larger change in the imaginary part of the dielectric permittivity, ${\varepsilon }_{r}^{"}$, compared to NiO, Fig. 16. The loss tangents in the non-actuated and actuated states for both NiO and TiO_2_ are shown in Figs. 17 and 18, respectively, which provide a more intuitive view on the dielectric behaviour of NiO and TiO_2_. Here, it is worth noting that the loss tangent of TiO_2_ in the non-actuated state is no less than 50 times lower than the corresponding loss tangent of NiO, as shown in Fig. 17 and considerably higher than that of NiO in the actuated state, as shown in Fig. 18. From Fig. 18 one can note that the loss tangent and, correspondingly from Fig. 16, the imaginary part of the dielectric permittivity, of TiO_2_ is extremely high. Such behavior is inherent in metals rather than dielectrics. In this case, it makes sense to evaluate material losses in the actuated (ON) states based on conductivity values, which can be extracted using the following formula (11):

11 $$\sigma = {\varepsilon }_{r}^{"}\omega {\varepsilon }_{0}.$$

**Figure 19:**
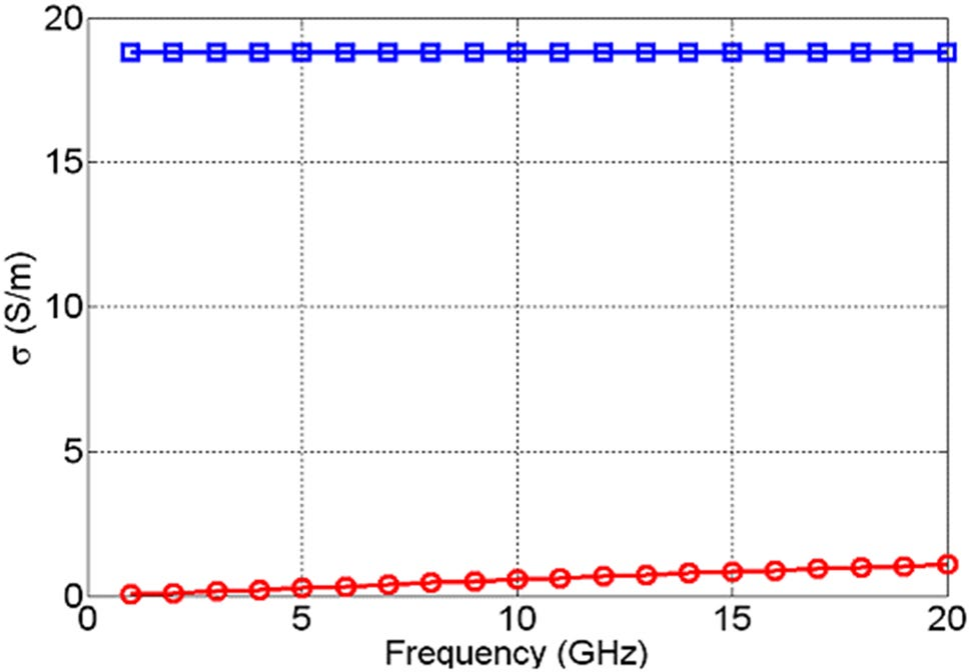
Extracted conductivities of NiO in ON state (red circles) and TiO_2_ in ON state (blue squares).

These results are shown in Fig. 19. As one can see, TiO_2_ outperforms NiO in the actuated, metallic state, too. Here, even though the conductivity of NiO increases as a function of frequency, it is still approximately at least 20 times lower that the conductivity of TiO_2_. It is interesting to see that the conductivity of TiO_2_ is constant as a function of frequency, while the conductivity of NiO monotonously increases as a function of frequency. This indicates that the switching mechanism is probably of a different character for the examined TMOs, which requires future experimental and theoretical investigation. The results presented indicate that even in the amorphous states, TMOs exhibit a phase transition (switching), extending up to 20 GHz. Even though the practical use of NiO as a reconfigurable material may be of limited use, TiO_2_, on the other hand, displays great potential. In the absence of DC bias voltage, it behaves as a good dielectric with loss tangents of the order of 3 × 10^–4^. However, upon DC bias voltage actuation, it transitions into a metallic state with the real part of the dielectric permittivity of around 110 and conductivity of around 20 S/m. Such a level of change in the constituent parameters in TiO_2_ is sufficient for many RF/mm-wave applications, such as phase shifters, attenuators, frequency tuneable antennas and filters, to name but a few. The results are extremely encouraging and point to new ways of achieving reconfigurability, which is of high importance to the currently deployable 5G technologies and the forthcoming 6G communication systems. In the next section, an example of RF device enabled by the examined switching behaviour of TiO_2_ are presented.

## RF switch and attenuator

**Figure 20:**
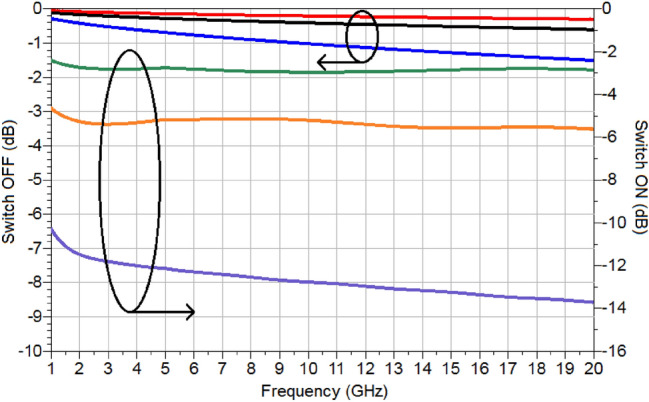
Performance of TiO_2_ based switch as function of length; red, black and blue—OFF state for lengths of 1 mm, 2 mm and 5 mm respectively and green, orange and purple—ON state corresponding to the same lengths.

Prior to examining the use of a TMO-based RF switch in a circuit, the performance of the switch needs to be evaluated. For a switch using TiO_2_, as elaborated in the previous section, its performance is a function of the length of the active region. To investigate this effect, the thicknesses of the bottom ground and top microstrip line are increased to 3 µm, so that the switch formed in this way does not suffer sub-skin depth losses. The length dependent performance of the TiO_2_ switch is shown in Fig. 20. As can be seen, it is a strong function of length—greater lengths incur a greater dynamic range, but, at the same time, have greater losses.

The performances of the switches at 15 GHz for several different lengths are shown in Table 1.

**Table 1:** Summary of the performance of the switch at 15 GHz.

Length (mm)	Freq. (GHz)	Insertion loss (dB)	Isolation (dB)	Dynamic range (dB)
1	15	0.25	2.84	2.59
2	15	0.51	5.56	5.05
5	15	1.27	13.24	11.97
10	15	2.55	26.2	23.65

**Figure 21:**
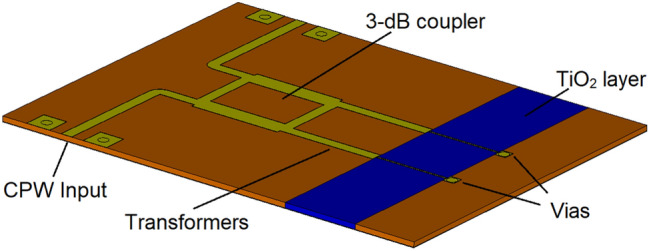
Reflective-type variable attenuator (RTVA) based on TiO_2_.

**Figure 22:**
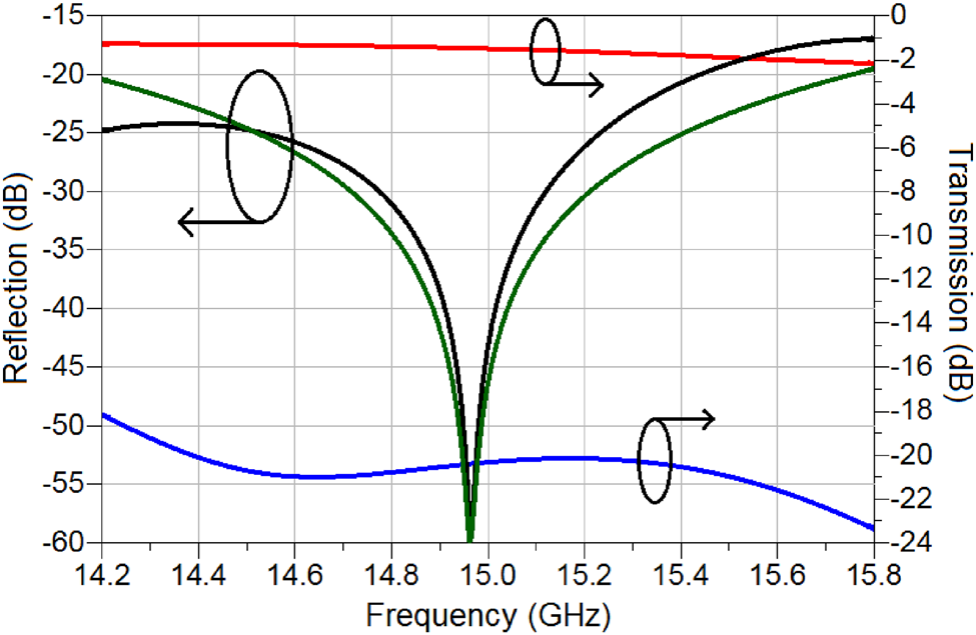
Performance of RTVA of figure, based on extracted values of TiO_2_; black—reflection coefficient in the OFF state, green—reflection coefficient in the ON state, red—insertion loss in the OFF state and blue—insertion loss in the ON state.

Next, based on the switching characteristics of TiO_2_ recorded in Fig. 20, a variable attenuator is designed, and its performance investigated through simulations. For the purpose of demonstration, a Reflective-Type Variable Attenuator (RTVA), based on a 3-dB coupler and reflective loads is used, Fig. 21. The 3-dB coupler is made to operate at a centre frequency of 15 GHz and was designed on a substrate (Roger Duroid, 3003) with ${\varepsilon }_{r}=3$ and tan(δ) = 0.001^30^. The substrate thickness is *h* = 130 µm. The reflective loads of the designed coupler, as shown in Fig. 21, consist of two sections. The first section has an active TiO_2_ region upon which a microstrip line is deposited and pertinently short circuited to the ground through a via. The physical length of this section is 3 mm. The active TiO_2_ region is connected to the 3-dB coupler through a quarter-wave transformer in order to extend the bandwidth of the operation of the proposed RTVA (Reflective-Type variable Attenuator). The characteristic impedance of the transformer is 50 Ω and its length corresponds to 3.16 mm. The dimensions of the entire RTVA structure of Fig. 21 are 15.18 mm × 11.77 mm × 0.13 mm. The response of the RTVA formed in this way is shown in Fig. 22. In the frequency window of 14.2–15.8 GHz, insertion losses vary from 1.2 to 2.1 dB, while the maximum values of attenuation are, in the same frequency range, 18.1 dB and 23.3 dB, respectively. As such, the minimum dynamic range offered by the present RTVA is around 17 dB. As mentioned previously, the dynamic range can be increased, however, that has the consequence of increasing insertion losses. In any case, there exists tremendous possibility to tailor the response of RTVA to suit a host of applications, depending on the needs, as a function of the length of the active region. The versatile nature of TMOs and in the present case TiO_2_ strongly requires further investigation. Our results on the switching characteristics of TiO_2_ show that a TMO does not the need to be in a “perfect” crystalline state in order to exhibit good RF/mm-wave switching characteristics, rather they point to the fact that even amorphous TMOs can display attractive RF/mm-wave characteristics. A major research question to answer lies with the possibility of tailoring the RF/mm-wave switching characteristics of amorphous TMOs by virtue of controlling the level of “defects” or impurities as alluded to in the introduction. This will be an exciting time for TMO research.

## Conclusions

In this paper, resistive switching of amorphous NiO and TiO_2_ TMOs was experimentally investigated for switched signals ranging up to frequencies of 20 GHz. The samples were fabricated using standard microfabrication techniques. The measurements indicate that TiO_2_ possesses superior electrical performance both in the non-actuated and actuated states, making this material highly promising for a variety of RF/mm-wave applications. Our study shows that in the non-actuated state, TiO_2_ is a dielectric with a dielectric permittivity of about 54 and a loss tangent in the region of 3 × 10^–4^. Upon activation, the material exhibits a complex insulator to metal transition, with an increase in the dielectric permittivity to over 110 and a corresponding conductivity of about 20 S/m. Such changes in the properties are in many cases adequate to support a variety of RF/mm-wave devices, such as phase shifters, attenuators, frequency tuneable oscillators and filters, to name but a few. As an example, a simulation study of a Reflective-Type Variable Attenuator (RTVA) based on TiO_2_ has demonstrated the potential of TMOs for realization of reconfigurable devices, where a dynamic range of 17 dB was recorded with insertion losses of only 1.2 dB. These levels of reconfigurability achieved so far are extremely promising and further research work is necessary to fully unlock their potential.

## Data Availability

The datasets used and/or analysed during the current study available from the corresponding author on reasonable request.
